# Risk-Association of DNA Methyltransferases Polymorphisms with Gastric Cancer in the Southern Chinese Population

**DOI:** 10.3390/ijms13078364

**Published:** 2012-07-05

**Authors:** Xue-Xi Yang, Xuan-Qiu He, Fen-Xia Li, Ying-Song Wu, Yang Gao, Ming Li

**Affiliations:** 1 School of Biotechnology, Southern Medical University, Guangzhou 510515, China; E-Mails: yxxzb@sohu.com (X.-X.Y.); lifenxia123@gmail.com (F.-X.L.); yingsongwu@hotmail.com (Y.-S.W.); jackey1976@126.com (Y.G.); 2 The First Clinical College, Southern Medical University, Guangzhou 510515, China; E-Mail: lmsh815@163.com

**Keywords:** gastric cancer (GC), *DNMTs*, single nucleotide polymorphism (SNP), susceptibility

## Abstract

DNA hypomethylation and/or hypermethylation are presumed to be early events in carcinogenesis, and one or more DNA methyltransferases (DNMTs) have been suggested to play roles in carcinogenesis of gastric cancer (GC). However, there have been no systematic studies regarding the association between *DNMT* gene polymorphisms and GC risk. Here, we examined the associations of 16 single nucleotide polymorphisms (SNPs) from *DNMT1* (rs2114724, rs2228611, rs2228612, rs8101866, rs16999593), *DNMT2* (rs11695471, rs11254413), *DNMT3A* (rs1550117, rs11887120, rs13420827, rs13428812, rs6733301), *DNMT3B* (rs2424908, rs2424913, rs6087990) and *DNMT3L* (rs113593938) with GC in the Southern Chinese population. We assessed the associations of these 16 SNPs with GC in a case-control study that consisted of 242 GC cases and 294 controls, using the Sequenom MALDI-TOF-MS platform. Association analyses based on the χ^2^ test and binary logistic regression were performed to determine the odds ratio (OR) and 95% confidence interval (95%CI) for each SNP. We found that rs16999593 in *DNMT1*, rs11254413 in *DNMT2* and rs13420827 in *DNMT3A* were significantly associated with GC susceptibility (OR 1.45, 0.15, 0.66, respectively; 95% CI 1.00–2.11, *p* = 0.047; 0.08–0.27, *p* < 0.01; 0.45–0.97, *p* = 0.034, respectively, overdominant model). These results suggested that *DNMT1*, *DNMT2* and *DNMT3A* may play important roles in GC carcinogenesis. However, further studies are required to elucidate the mechanism.

## 1. Introduction

Gastric cancer (GC) is one of the most common malignant tumors in China with a high incidence and mortality rate [1,2]. GC is a complex disease as its pathogenesis includes the interaction between environmental exposure and genetic variations, which could result either directly or indirectly in systemic epigenetic control defects. Epigenetic events or a stably heritable phenotype in gene expression capacity through chromosome modifications without DNA sequence alterations have been studied to gain insight into the aetiology of the disease [3], and the results have focused attention on the links between epigenetic pathways and tumor initiation and progression. The most commonly studied epigenetic phenomenon is DNA methylation, an essential regulator of transcription and chromatin structure. In mammals, this mainly refers to the covalent post-replicative addition of a methyl group to the 5′ position of a cytosine in a CpG dinucleotide [4], which is conferred by DNA methyltransferases (DNMTs). DNMTs can be classified into three families: DNMT1, DNMT2 and DNMT3 (DNMT3A and DNMT3B) [5]. DNMT1 acts primarily as a maintenance methyltransferase at each cell division [6]. DNMT2, also named TRDMT1 (tRNA aspartic acid methyltransferase), is distinct and highly conserved among taxa, and mediates these activities in both DNA and RNA methylation [7]. DNMT3A and DNMT3B are considered to be *de novo* DNA methyltransferases during gametogenesis and embryogenesis [8,9]. DNMT3L methylation is often referred to as stimulating *de novo* methylation machinery by interacting with the catalytic domains of DNMT3A and DNMT3B [10].

The family of DNMTs provides a mechanism for the stable and heritable silencing of transcription, and plays a crucial role in the way that mammalian genomes are structurally organized, functionally regulated and perpetually maintained. It has been reported that DNMTs are essential for proper embryogenesis [11] and the formation of mature functional germ cells [12,13]. Likewise, aberrant DNA methylation patterns in a genetically susceptible background may be associated with increased risk of a series of human disorders [14–16], including cancer, such as GC [17]. In a systematic search, El-Maarri *et al.* demonstrated the polymorphisms in all coding regions of the five *DNMT* genes and confirmed the significant association between a rare *DNMT3L* variant (R271Q, *i.e.*, rs113593938) and subtelomeric hypomethylation [18].

Most previous reports have focused on three functional enzymes, DNMT1, DNMT3A and DNMT3B [8,19], and there have been some studies of polymorphisms in these three *DNMTs* associated with GC [20–24]. However, the associations between these polymorphisms and clinical implications of GC are still uncertain. There have been few studies of the possible association of cancer susceptibility with the other DNMTs, such as DNMT2 and DNMT3L. In addition, the prevalence of *DNMT* SNPs in the southern Chinese population has not been documented. The present study was performed to evaluate whether the 16 SNPs in different *DNMTs* among 242 GC cases and 294 frequency-matched unaffected controls were associated with cancer susceptibility.

## 2. Results and Discussion

### 2.1. Subject Characteristics

A total of 242 GC cases and 294 controls were included in this study, and the clinical characteristics of the cases and controls are detailed in Table 1. Males accounted for 71.9% of the cases compared with 59.5% of the controls. The mean ± SD age (at diagnosis) of the patients was 54.9 ± 12.5 years (range 21–79 years), and that of the controls was 58.4 ± 16.4 years (range 14–94 years). The distribution of gender and mean age between cases and controls indicated significant difference, so all statistical analyses were subsequently adjusted by sex and age. Of 242 patients, the most common specified sites were in cardia or spanned over two anatomical regions of the stomach, accounting for 90.1% of cases. A total of 63 patients (26.1%) were classified as well or moderately differentiated GC, 132 (54.5%) poorly differentiated GC and 19 (7.8%) signet-ring cell carcinomas, while the remaining 28 patients (11.6%) were unclassified. The cases consisted of 68 (28.1%) in the clinical stage I or II groups and 160 (71.9%) in the clinical stage III or IV groups. Only 26 patients (10.7%) were present in the early gastric cancer group, in which the depth invasion is confined to lamina propria or submucosa, 89.2% of patients were found to have advanced GC including T2, T3 and T4. For gastric cancer, infection with *Helicobacter pylori* is the main etiologic factor. However, infection with *Helicobacter pylori* had not received enough attention in gastric cancer until several years ago in China. Among these collected samples, patients collected in 2009 were not checked for the *H. pylori* infections, however, patients collected in 2010 were examined for the *H. pylori* infection. This leads to about 66% percent of samples that have lost the data of *H. pylori* infection. Therefore, information regarding infection with *Helicobacter pylori* was not present.

### 2.2. Genotype Information

Sixteen SNPs in *DNMTs* were chosen from the public single nucleotide polymorphism database dbSNP [25], *i.e.*, rs2114724, rs2228611, rs2228612, rs8101866 and rs16999593 in *DNMT1*; rs11695471 and rs11254413 in *DNMT2*; rs1550117, rs11887120, rs13420827, rs13428812 and rs6733301 in *DNMT3A*; rs2424908, rs2424913 and rs6087990 in *DNMT3B*; and rs113593938 in *DNMT3L*. Table 2 shows detailed information regarding these 16 SNPs.

Among these 16 SNPs, rs6087990 and rs11695471 had call rates <80%, and one SNP (rs2228612) showed deviation from the Hardy–Weinberg equilibrium expectations in the control population (*p* > 0.05). In addition, three SNPs (rs2424913, rs6733301 and rs113593938) were non-polymorphic in this study, with minor allele frequencies (MAF) of less than 0.01. The major genotypes were TT, GG and CC for the three SNPs rs2424913, rs6733301 and rs113593938, respectively. The above-mentioned six SNPs were excluded from subsequent analyses. The remaining 10 SNPs—consisting of four in *DNMT1*, one in *DNMT2*, four in *DNMT3A* and one in *DNMT3B*—were successfully genotyped.

### 2.3. MAF of the Chosen SNPs with HapMap Data

The MAFs of the 10 SNPs investigated are summarized in Table 3. The MAFs in our study showed greater similarities with Beijing and Japanese populations, and greater differences with European and African populations. Moreover, there was a significant difference between cases and controls in our study for rs11254413 in *DNMT2* (*p* < 0.01). Logistic regression analysis, adjusted for age and sex, showed the A allele of SNP rs11254413 with an odds ratio (OR) of 0.65 (95% confidence interval (95% CI): 0.48–0.87) for GC. However, no statistically significant associations were found between any of the other SNPs and GC.

### 2.4. Associations between Individual SNPs and GC Risk

Five genetic models (co-dominant, dominant, recessive, over-dominant, and log-additive) were chosen to evaluate the association between each SNP and GC risk. Genotype frequencies of cases and controls as well as OR and *p* values for the best-fitting genetic model (over-dominant) and co-dominant model are shown in Table 4. Three other models (dominant, recessive, and log-additive) are presented in Table S1. Significant associations between three of the 10 genotyped SNPs (rs1699593, rs11254413, rs13420827) and GC were confirmed.

CT heterozygosity in rs1699593 in *DNMT1* was shown to be a risk factor for GC (*p* = 0.05 for overdominant model of SNPs analyses) with OR = 1.45 (1.00–2.11). A protective effect against GC was identified for AG heterozygosity of rs11254413 in *DNMT2* (*p* < 0.01 for overdominant model) with OR = 0.15 (0.08–0.27). In addition, there was a significant protective effect between GC heterozygosity in rs13420827 in *DNMT3A* and GC in the overdominant model (*p* = 0.034), with OR = 0.66 (0.45–0.97). No significant associations with GC susceptibility between cases and controls were found for other SNPs, including rs2114724, rs2228611, rs8101866 in *DNMT1*, rs1550117, rs11887120 and rs13428812 in *DNMT3A*, and rs2424908 in *DNMT3B* (see Table 4).

### 2.5. Linkage Disequilibrium of the SNPs in DNMT1 and DNMT3A

**Figure 1:**
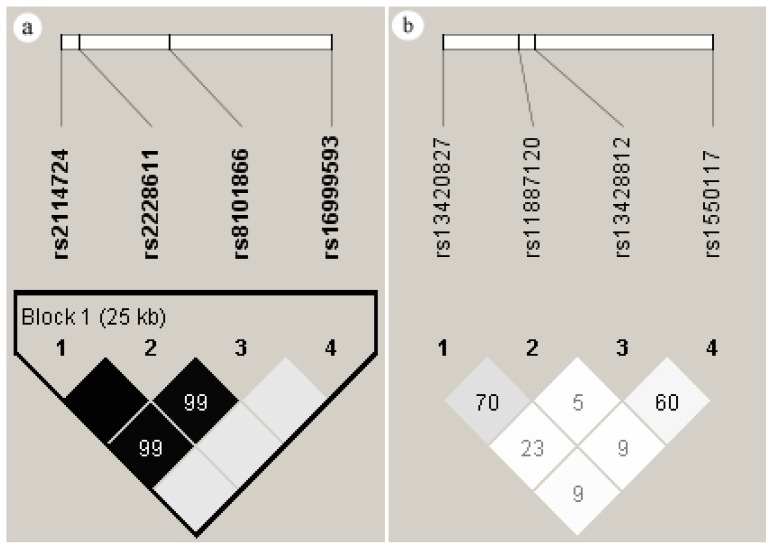
Linkage disequilibrium (LD) pattern of *DNMT1* (**a**) and *DNMT3A* (**b**) SNPs by Haploview analysis. Numbers inside the boxes represent *r*^2^ values for LD, and miss numbers were 100. Shaded regions in the LD plot indicate the strength of LD between pair wise combinations of SNPs (white, low LD; black, high LD).

Four SNPs in *DNMT1* and 4 SNPs in *DNMT3A* were successfully genotyped. Linkage disequilibria for these SNPs in the *DNMT1* and *DNMT3A* genes are shown in Figure 1. The four SNPs in *DNMT1* formed a block within 25 kb (chr: 10265248–10291181), whereas the four in *DNMT3A* did not (chr: 25453968–25565907). The D′ and *r*^2^ values between SNPs are presented in Table S2. Due to the strong linkage in *DNMT1*, tagged SNPs were run in Haploview. The results indicated that rs8101866 and rs16999593 could act as tag SNPs for the four SNPs in *DNMT1*. The SNP rs8101866 had higher *r*^2^ with rs2114724 and rs2228611 (0.976 and 0.97, respectively).

### 2.6. Haplotypes of the SNPs in DNMT1 and DNMT3A

The haplotypes of *DNMT1* and *DNMT3A* using global haplotype score tests for association with GC risk are shown in Table 5. Of the three individual haplotypes estimated in *DNMT1*, the most frequent haplotype CGTT was seen in 53.9% of the controls and 51.0% of the patients. The most frequent *DNMT3A* haplotype in patients and controls was CCAG (25.5% and 27.6%, respectively). There were no significant differences in haplotype frequency distribution between GC patients and controls for any of the haplotypes examined.

Three of the 16 SNPs examined showed significant associations with GC, *i.e.*, a missense mutation of rs16999593 in *DNMT1* (His97Arg substitution), a missense mutation of rs11254413 in *DNMT2* (His101Tyr substitution) and a synonymous mutation of rs13420827 in *DNMT3A*. Previous studies indicated associations of rs16999593 with risk of infiltrating ductal breast carcinoma (IDC) [26] in the northern Chinese population (Heilongjiang Province), and of rs13420827 with ovarian cancer [27,28] in the American population (Mayo and North Carolina). However, no association between gastric cancer and *DNMT* polymorphisms among southern Chinese patients has been reported. To our knowledge, this is the first report of the association of these three gene variants with GC susceptibility.

Abnormal DNA methylation is thought to be a major early event in tumor development. For example, tumor suppressor genes are silenced by DNA methylation on CpG islands around their promoter regions in cancer cells, and thus the overall level of DNA methylation is higher in cancer cells than in normal cells [29,30]. SNPs of *DNMTs* are important indicators of genetic susceptibility to cancer development. Therefore, genetic polymorphism assays have been used to investigate the aetiology of malignant diseases [31].

In our case-control study, four of five SNPs in the *DNMT1* gene were successfully genotyped (rs2228612 failed due to Hardy–Weinberg equilibrium deviation) and only rs16999593 was significantly associated with GC. CT heterozygosity in rs16999593 was associated with an increased risk of GC; C→T variation results in an Arg to His amino acid substitution in *DNMT1. DNMT1* is located at the replication fork and methylates newly biosynthesised DNA strands immediately after the round of replication [32]. Therefore, the variation leading to missense mutation in rs1699593 may affect the structure and function of DNMT1. Inactivation of *DNMT1* has been shown to lead to mitotic catastrophe in human cancer cells [33]. Previous studies indicated that the level of *DNMT1* expression is significantly elevated in GC, and suggested that *DNMT1* can be used as a predictive biomarker and potential therapeutic target for chemotherapy in GC [34–36]. Increased DNMT1 protein expression is significantly correlated with poorer tumor differentiation and frequent DNA hypermethylation of multiple CpG islands in GC [37]. However, Khatami *et al.* [21] found no associations between GC susceptibility and *DNMT1* polymorphisms, including rs721186, rs13784, rs2228611 and rs11488, in an Iranian population. Based on the haplotype analysis in the present study, we could not confirm associations between four SNPs in *DNMT1* and GC risk, while a previous study indicated that the CA haplotype (rs16999593 T, rs2228611 G) may be a risk factor for breast IDC [26]. With regard to the pivotal role of methylation in the *DNMT1* promoter in tumourigenesis, we hypothesized that CT heterozygosity in rs16999593 could contribute to genetic susceptibility to GC.

The AG genotype of rs11254413 in *DNMT2* had a lower frequency in patients than in controls. The SNP rs11254413 leads to missense mutation of His to Tyr, and may also alter the function of the protein encoded by the *DNMT2* gene. DNMT2 is primarily localized to the cytoplasm [38] and is responsible for the methylation of aspartic acid tRNA and possesses residual DNA (cytosine-C5) methyltransferase activity [39]. Although it is the most widely conserved DNMT protein, little is known about DNMT2 compared to other DNMT proteins. El-Maarri *et al.* reported that rs11254413 was associated with hypermethylation at a small subset of loci, but it was unclear whether this SNP *per se* played a causative role [18]. The results of the present study indicate a potentially protective effect of AG heterozygosity of rs11254413 on GC risk. Therefore, these results further emphasize the importance of defining DNMT2-modulated cellular pathways in future studies.

*DNMT3A* and *DNMT3B* are localized on chromosomes 2p23 and 20q11.2, respectively. As *de novo* DNA methyltransferases, they play important roles in the generation of aberrant methylation in carcinogenesis. In gastric cancers, CpG island hypermethylation of tumour suppressor genes are more frequently inactivated by aberrant DNA methylation [40–42]. The polymorphism of rs1550117 in *DNMT3A* has been reported to be associated with susceptibility to GC among the Chinese population in Jiangsu province [22]. In the present study, we detected a possible association of rs13420827 but not rs1550117 with GC susceptibility. Thus, the potential association between missense mutation of rs13420827 and the change in DNMT3A activity remains uncertain. Kelemen *et al.* reported that rs13420827 was not associated with ovarian carcinoma [27].

With regard to *DNMT3B* polymorphisms, previous studies focused on the transcriptional start site within the promoter region, especially the SNPs rs2424913 and rs1569686. Hu *et al.* [23] reported associations of rs2424913 and rs1569686 with GC in a Chinese population. In the present study, the frequency of allele C on rs2424913 was lower than 0.01, which was consistent with reports from Japan and Hebei, China [24,43]. In addition, we found no significant differences in rs1569686 and rs2424908, although these three SNPs have been reported to be related to other types of tumor, such as oesophageal cancer, breast cancer, colorectal cancer, *etc*. [44–46].

DNMT3L is an inactive accessory factor among the DNMTs. It cannot directly methylate DNA as it lacks the amino acid residues necessary for methyltransferase activity [47]. However, DNMT3L acts as a regulator of *de novo* methyltransferases by stimulating the activities of DNMT3A and DNMT3B through protein–protein interactions [48]. In addition, DNMT3L is important for embryonic development, imprinting and X-chromosome inactivation, which may lead to the genome-wide nuclear reprogramming observed in tumor cells [49]. With regard to rs113593938 in *DNMT3L*, the C→T allele leads to the missense mutation of Arg to Gln. While the locus lacked gene polymorphisms among the southern Chinese population in the present study, the distribution of CC was 100%, in accordance with the data from dbSNP in a cohort population [25]. Recent reports have suggested that hypomethylation in the *DNMT3L* promoter may be a novel marker of cervical cancer [50]. However, few association studies of *DNMT3L* polymorphisms have been reported, with the exception of that between rs7354779 and human intelligence [51].

## 3. Experimental Section

### 3.1. Subjects

All subjects included in the present case-control study were recruited from the outpatient and inpatient clinics of the First Affiliated Hospital of Nanchang University, Nanchang, Jiangxi Province, China, from 2009 to 2010. Patients selected for the study were those with histologically-confirmed GC. Controls were randomly selected cancer-free individuals. The collection of samples and their utilization for research purposes were approved by the Southern Medical University Ethics Committee, and all subjects provided written informed consent prior to enrolment.

Peripheral blood samples were drawn from participants at the First Affiliated Hospital of Nanchang University. The samples were delivered in the frozen state by express mail to the School of Biotechnology, Southern Medical University, and stored at −70 °C until DNA extraction. Genomic DNA was extracted using a commercial blood DNA kit (TIANamp Genomic DNA Purification Kit; Tiangen Biotech, Beijing, China), according to the manufacturer’s instructions, and stored at −70 °C until use.

### 3.2. SNP Selection and Genotyping

The inclusion criteria of candidate SNPs selection are SNPs among *DNMT* genes (*DNMT1*, *DNMT2*, *DNMT3A*, *DNMT3B* and *DNMT3L*) and previous literatures [18,21–24] have been reported the association with cancers, especially gastric cancer; SNPs data from the Han Chinese Population included in the HapMap, were also used to as referred [52]. Furthermore, SNPs failed in assay design were excluded. A total of 16 frequency-validated SNPs were selected for genotyping.

These 16 SNPs were genotyped using the SEQUENOM MassARRAY matrix-assisted laser desorption ionisation-time of flight mass spectrometry platform (Sequenom, San Diego, CA, USA). Primers for multiplex PCR and extended reactions were designed using proprietary software (Assay Designer, version 3.1; Sequenom: San Diego, CA, USA, 2006) provided by Sequenom Inc. In accordance with the manufacturer’s instructions, the first step was to amplify the genomic sequence containing the SNP by a standard PCR protocol, which would produce amplicons 80–120 bp in length. Excess PCR primers and unincorporated dNTPs were neutralized by incubation with shrimp alkaline phosphatase (SAP). Subsequently, the single-base extension (SBE) reaction was performed on the genomic amplification product using iPLEX enzyme and mass-modified terminators. The process was designed to generate SNP-specified DNA products of different lengths with predictable masses. The products of the iPLEX reaction were desalted and transferred onto a SpectroCHIP by the MassARRAY nanodispenser. The SpectroCHIP was then analyzed by the MassARRAY Analyzer Compact.

### 3.3. Data Quality Assessment and Statistical Analysis

The genotypes were examined separately by a detailed quality control (QC) process. The SNP genotype threshold was set at 80%. SNPs not in Hardy–Weinberg equilibrium (*p* < 0.05) in the control samples were excluded from all analyses. SNPs with MAF ≤ 0.01 were also excluded from the subsequent analyses.

The results are expressed as proportions or means and standard deviation (SD). Differences in genotypes and alleles between GC patients and healthy controls were assessed using the χ^2^ test. The associations between GC and individual *DNMT* SNPs were estimated by logistic regression with adjustment for age and sex. Statistical analyses and haplotyping were performed using the web-based tool SNPstats [53]. Five genetic models (co-dominant, dominant, recessive, over-dominant, and log-additive) were chosen to evaluate the association between each SNP and GC risk. Two alternative models (co-dominant and overdominant) were fitted for each outcome, adjusting for age and gender as covariates. The linkage disequilibrium (LD) and pairwise LD coefficients in *DNMT1* and *DNMT3A* were implemented with Haploview 4.2 (Daly Lab: Cambridge, MA, USA, 2008). All comparisons were two tailed, and *p* < 0.05 was considered to indicate statistical significance.

## 4. Conclusions

In the present study, we evaluated 16 SNPs in an independent population and showed that polymorphisms of three genetic variants, defined by rs16999593 in *DNMT1*, rs11254413 in *DNMT2* and rs13420827 in *DNMT3A*, were associated with GC susceptibility in a southern Chinese population. Although the other 13 SNPs failed to show significant associations with GC in the present population, these results suggest that *DNMTs* are involved in gastric carcinogenesis. However, the present study was carried out in a relatively small population with less than 300 cases or controls, furthermore, after multiple comparison adjustment, only rs11254413 in *DNMT2* still showed significant association. Additional studies using a larger sample size are required to confirm our findings. To elucidate the true effects of *DNMT* polymorphisms in determining the pathogenesis of GC, investigations of other variants and their influence on the biological functions of DNMTs are also required.

## Supplementary Information



## Figures and Tables

**Table 1:** Characteristics of study subjects.

Variable		Cases (*n* = 242) N (%)	Controls (*n* = 294) N (%)	*p*
Sex	Male	174 (71.9)	175 (59.5)	0.03 a
	Female	68 (28.1)	119 (40.5)	-
Mean (SD)		54.9 (12.5)	58.4 (16.4)	0.005 b
Age (years)	≤60	155 (64.0)	146 (49.7)	0.179
	>60	87 (36.0)	148 (50.3)	-
Tumor sites	Non-cardiac	24 (9.9)	-	-
	Cardiac	218 (90.1)	-	-
Histological types	Well	21 (8.7)	-	-
	Moderate	42 (17.4)	-	-
	Poor	132 (54.5)	-	-
	Signet ring cell	19 (7.8)	-	-
	Unclassified	28 (11.6)	-	-
Clinical stage (TNM)	I	19 (7.9)	-	-
	II	49 (20.2)	-	-
	III	119 (49.2)	-	-
	IV	55 (22.7)	-	-
Depth of invasion c	T1	26 (10.7)	-	-
	T2	64 (26.4)	-	-
	T3	104 (43.0)	-	-
	T4	48 (19.8)	-	-

a Two-sided test χ^2^;

b T test;

c T1 Lamina propria or submucosa; T2 Muscularis propria or subserosa; T3 Serosa (visceral peritoneum) without invasion of adjacent structures; T4 Adjacent structures.

**Table 2:** Chosen single nucleotide polymorphisms (SNPs) from DNA methyltransferase (DNMT)s.

No.	SNP	Gene	Location	From	To	Change	Codon
1	rs2114724	*DNMT1*	Intron	G	A	10265248A>G	—
2	rs2228611	*DNMT1*	Coding region	G	A	Pro463Pro	463
3	rs2228612	*DNMT1*	Coding region	A	C/G/T	Ile327Leu,Phe,Val	327
4	rs8101866	*DNMT1*	Intron	A	G	10275660G>A	—
5	rs16999593	*DNMT1*	Coding region	T	C	His97Arg	97
6	rs11254413	*DNMT2*	Coding region	A	G	His101Tyr	101
7	rs6733301	*DNMT3A*	Intron	G	A	25276284G>A	—
8	rs13420827	*DNMT3A*	3′ untranslated region	G	C	25453968C>G	—
9	rs11695471	*DNMT3A*	Intron	T	A	25457708T>A	—
10	rs11887120	*DNMT3A*	Intron	T	A/C/G	25485735G>T,C,A	—
11	rs13428812	*DNMT3A*	Intron	A	G	25492467A>G	—
12	rs1550117	*DNMT3A*	5′ near gene	G	A	25565907A>G	—
13	rs6087990	*DNMT3B*	5′ near gene	T	C	31349908T>C	—
14	rs2424908	*DNMT3B*	Intron	C	T	31360383C>T	—
15	rs2424913	*DNMT3B*	Intron	C	T	31374259C>T	—
16	rs113593938	*DNMT3L*	Coding region	C	T	Arg271Gln	271

**Table 3:** Interethnic comparisons of minor allele frequencies for 10 SNPs in our subjects with HapMap data.

Genes/SNP	Minor Allele	Present Study		HapMap Data			
	
		Control	GC	Beijing	Japan	European	African
*DNMT1*							
rs2114724	T	0.26	0.25	0.27	0.38	0.51	0.53
rs2228611	A	0.26	0.25	0.27	0.39	0.51	0.53
rs8101866	C	0.19	0.23	0.27	0.44	0.53	0.52
rs16999593	C	0.26	0.26	0.17	0.2	0.00	0.00
*DNMT2*							
rs11254413	A	0.21	0.13	0.28	0.23	0.48	0.42
*DNMT3A*							
rs1550117	A	0.19	0.2	0.16	0.13	0.17	0.18
rs11887120	C	0.48	0.51	0.52	0.51	0.61	0.54
rs13420827	G	0.2	0.18	0.24	0.3	0.3	0.33
rs13428812	G	0.26	0.26	0.15	0.22	0.08	0.05
*DNMT3B*							
rs2424908	C	0.43	0.44	0.5	0.57	0.81	0.64

**Table 4:** Odds ratio (OR) for case-control study of 10 gastric cancer (GC) susceptibility loci.

Genes/SNP	Genotype	Cases N (%)	Controls N (%)	Co-Dominant Model		Overdominant Model	

				OR (95% CI)	*p* a	OR (95% CI)	*p* b
*DNMT1*							
rs2114724	CC	132 (54.5)	162 (56.2)	1.00	0.22	1.00	0.27
	CT	97 (40.1)	101 (35.1)	1.16 (0.81–1.68)	-	1.23 (0.86–1.76)	-
	TT	13 (5.4)	25 (8.7)	0.62 (0.30–1.27)	-	-	-
rs2228611	GG	132 (54.5)	160 (56.1)	1.00	0.14	1.00	0.22
	AG	97 (40.1)	99 (34.7)	1.18 (0.81–1.71)	-	1.26 (0.87–1.80)	-
	AA	13 (5.4)	26 (9.1)	0.58 (0.28–1.18)	-	-	-
rs8101866	TT	130 (53.9)	166 (56.5)	1.00	0.17	1.00	0.19
	CT	98 (40.7)	102 (34.7)	1.20 (0.83–1.74)	-	1.27 (0.89–1.82)	-
	CC	13 (5.4)	26 (8.8)	0.61 (0.30–1.26)	-	-	-
rs16999593	TT	141 (58.3)	196 (66.7)	1.00	0.13	1.00	**0.05**
	CT	89 (36.8)	83 (28.2)	**1.47 (1.01**–**2.14)**	-	**1.45 (1.00**–**2.11)**	-
	CC	12 (5.0)	15 (5.1)	1.14 (0.51–2.54)	-	-	-
*DNMT2*							
rs11254413	GG	204 (84.3)	187 (63.8)	1.00	<0.0001	1.00	**<0.0001**
	AG	15 (6.2)	91 (31.1)	**0.16 (0.09**–**0.28)**	-	**0.15 (0.08**–**0.27)**	-
	AA	23 (9.5)	15 (5.1)	1.45 (0.73–2.90)	-	-	-
*DNMT3A*							
rs1550117	GG	157 (64.9)	191 (65.0)	1.00	0.74	1.00	0.68
	AG	74 (30.6)	93 (31.6)	0.94 (0.64–1.37)	-	0.92 (0.63–1.34)	-
	AA	11 (4.5)	10 (3.4)	1.34 (0.55–3.29)	-	-	-
rs11887120	TT	57 (23.6)	74 (25.3)	1.00	0.46	1.00	0.39
	CT	121 (50.0)	155 (53.1)	0.96 (0.63–1.47)	-	0.86 (0.61–1.22)	-
	CC	64 (26.4)	63 (21.6)	1.26 (0.76–2.07)	-	-	-
rs13420827	CC	167 (69.0)	183 (62.7)	1.00	**0.046**	1.00	**0.034**
	CG	61 (25.2)	99 (33.9)	0.68 (0.46–1.01)	-	**0.66 (0.45**–**0.97)**	-
	GG	14 (5.8)	10 (3.4)	1.75 (0.74–4.14)	-	-	-
rs13428812	AA	137 (56.6)	160 (55.4)	1.00	0.84	1.00	0.63
	AG	84 (34.7)	106 (36.7)	0.93 (0.64–1.35)	-	0.91 (0.64–1.31)	-
	GG	21 (8.7)	23 (8.0)	1.11 (0.58–2.12)	-	-	-
*DNMT3B*							
rs2424908	TT	78 (32.2)	99 (33.7)	1.00	0.96	1.00	0.82
	CT	114 (47.1)	139 (47.3)	0.98 (0.66–1.45)	-	0.96 (0.68–1.36)	-
	CC	50 (20.7)	56 (19.1)	1.05 (0.64–1.71)	-	-	-

Note: CI, confidence interval; OR, odds ratio; *p*-values obtained by chi-squared, OR (95% CI) was adjusted by sex and age; *p*

a for codominant model, *p*

b for overdominant model; Bold mean *p* < 0.05.

**Table 5:** Multivariable-adjusted haplotype analysis of *DNMT1* and *DNMT3A* genes and gastric cancer risk (*n* = 242).

Genes	Haplotype	Frequencies			OR * (95% CI)	*p* Value

		Total	Control	Case		
*DNMT1*	CGTT	0.526	0.539	0.510	1.00	-
	TACT	0.257	0.258	0.254	1.01 (0.76–1.36)	0.93
	CGTC	0.211	0.192	0.234	1.24 (0.91–1.70)	0.17
*DNMT3A*	CCAG	0.268	0.276	0.255	1.00	-
	CTAG	0.194	0.181	0.208	1.27 (0.80–2.01)	0.31
	CCGG	0.105	0.086	0.130	1.47 (0.82–2.63)	0.19

Haplotypes were constructed from the following SNPs: rs2114724, rs2228611, rs8101866, rs16999593 on *DNMT1*, and rs13420827, rs11887120, rs13428812, rs1550117 on *DNMT3A*; Only haplotypes with ≥0.10 frequencies;

* adjusted for age and sex.
