# Supplementary material for: Risk-Association of DNA Methyltransferases Polymorphisms with Gastric Cancer in the Southern Chinese Population
Source: Int J Mol Sci. 2012 Jul 5;13(7):8364–78. doi: 10.3390/ijms13078364 (PMC3430239; doi:10.3390/ijms13078364)
Supplement: Supplementary file 1 [file ijms-13-08364-s001.pdf]

Risk-Association of DNA Methyltransferases Polymorphisms with Gastric Cancer in the Southern Chinese Population

Supplementary Information

Table S1. OR for case-control study of 10 GC susceptibility loci.

SNP	Model	Genotype	Case n (%)	Control n (%)	OR (95% CI) [a]	*p*-value
DNMT1						
rs2114724	Dominant	C/C	132 (54.5)	162 (56.2)	1.00	0.77
		T/C-T/T	110 (45.5)	126 (43.8)	1.05 (0.74–1.50)	
	Recessive	C/C-T/C	229 (94.6)	263 (91.3)	1.00	0.13
		T/T	13 (5.4)	25 (8.7)	0.58 (0.29–1.18)	
	Log-additive	-	-	-	0.95 (0.72–1.25)	0.70
rs2228611	Dominant	G/G	132 (54.5)	160 (56.1)	1.00	0.78
		A/G-A/A	110 (45.5)	125 (43.9)	1.05 (0.74–1.49)	
	Recessive	G/G-A/G	229 (94.6)	259 (90.9)	1.00	0.08
		A/A	13 (5.4)	26 (9.1)	0.54 (0.27–1.09)	
	Log-additive	-	-	-	0.93 (0.71–1.23)	0.61
rs8101866	Dominant	T/T	130 (53.9)	166 (56.5)	1.00	0.65
		C/T-C/C	111 (46.1)	128 (43.5)	1.08 (0.76–1.53)	
	Recessive	T/T-C/T	228 (94.6)	268 (91.2)	1.00	0.11
		C/C	13 (5.4)	26 (8.8)	0.57 (0.28–1.15)	
	Log-additive	-	-	-	0.96 (0.73–1.26)	0.76
rs16999593	Dominant	T/T	141 (58.3)	196 (66.7)	1.00	0.055
		C/T-C/C	101 (41.7)	98 (33.3)	1.42 (0.99–2.03)	
	Recessive	T/T-C/T	230 (95.0)	279 (94.9)	1.00	1.00
		C/C	12 (5.0)	15 (5.1)	1.00 (0.45–2.21)	
	Log-additive	-	-	-	1.27 (0.94–1.70)	0.11
DNMT2						
rs11254413	Dominant	T/T	57 (23.6)	74 (25.3)	1.00	**<0.0001**
		C/T-C/C	185 (76.5)	218 (74.7)	**0.34 (0.22–0.52)**	
	Recessive	T/T-C/T	178 (73.5)	229 (78.4)	1.00	**0.045**
		C/C	64 (26.4)	63 (21.6)	**2.00 (1.01–3.98)**	
	Log-additive	-	-	-	**0.65 (0.48–0.87)**	**0.004**
DNMT3A						
rs1550117	Dominant	G/G	157 (64.9)	191 (65.0)	1.00	0.9
		A/G-A/A	85 (35.1)	103 (35.0)	0.98 (0.68–1.40)	
	Recessive	G/G-A/G	231 (95.5)	284 (96.6)	1.00	0.48
		A/A	11 (4.5)	10 (3.4)	1.37 (0.57–3.33)	
	Log-additive	-	-	-	1.02 (0.75–1.39)	0.89
rs11887120	Dominant	T/T	57 (23.6)	74 (25.3)	1.00	0.82
		C/T-C/C	185 (76.5)	218 (74.7)	1.05 (0.70–1.57)	
	Recessive	T/T-C/T	178 (73.5)	229 (78.4)	1.00	0.22
		C/C	64 (26.4)	63 (21.6)	1.29 (0.86–1.94)	
	Log-additive	-	-	-	1.12 (0.87–1.44)	0.37

Table S1. *Cont.*

SNP	Model	Genotype	Case n (%)	Control n (%)	OR (95% CI) [a]	*p*-value
rs13420827	Dominant	C/C	167 (69.0)	183 (62.7)	1.00	0.18
		G/C-G/G	75 (31.0)	109 (37.3)	0.78 (0.54–1.12)	
	Recessive	C/C-G/C	228 (94.2)	282 (96.6)	1.00	0.12
		G/G	14 (5.8)	10 (3.4)	1.97 (0.84–4.61)	
	Log-additive	-	-	-	0.92 (0.68–1.24)	0.58
rs13428812	Dominant	A/A	137 (56.6)	160 (55.4)	1.00	0.81
		G/A-G/G	105 (43.4)	129 (44.6)	0.96 (0.68–1.36)	
	Recessive	A/A-G/A	221 (91.3)	266 (92.0)	1.00	0.67
		A/A	21 (8.7)	23 (8.0)	1.14 (0.61–2.15)	
	Log-additive	-	-	-	1.00 (0.76–1.31)	1.00
DNMT3B						
rs2424908	Dominant	T/T	78 (32.2)	99 (33.7)	1.00	0.99
		C/T-C/C	164 (67.8)	195 (66.3)	1.00 (0.69–1.44)	
	Recessive	T/T-C/T	192 (79.3)	238 (81.0)	1.00	0.79
		C/C	50 (20.7)	56 (19.0)	1.06 (0.69–1.64)	
	Log-additive	-	-	-	1.02 (0.80–1.30)	0.89

Note: CI, confidence interval; OR, odds ratio; *p*-values obtained by chi-squared; [a] OR (95% CI) was adjusted by sex and age; Bold mean $p < 0.05$.

Table S2. The D' and r^2 value between SNPs in *DNMT1* and *DNMT3A*.

	L1	L2	D'	LOD	r^2
DNMT1					
	rs2114724	rs2228611	1.0	197.54	0.99
	rs2114724	rs8101866	0.99	190.58	0.976
	rs2114724	rs16999593	1.0	17.45	0.093
	rs2228611	rs8101866	0.99	187.91	0.97
	rs2228611	rs16999593	1.0	17.84	0.094
	rs8101866	rs16999593	1.0	17.96	0.094
DNMT3A					
	rs13420827	rs11887120	0.706	15.9	0.118
	rs13420827	rs13428812	0.233	0.56	0.005
	rs13420827	rs1550117	0.093	1.0	0.009
	rs11887120	rs13428812	0.057	0.13	0.001
	rs11887120	rs1550117	0.097	0.23	0.002
	rs13428812	rs1550117	0.604	4.25	0.032
